# Single Rooms, Seclusion and the Non-Restraint Movement in British Asylums, 1838–1844

**DOI:** 10.1093/shm/hky015

**Published:** 2018-03-14

**Authors:** Leslie Topp

**Affiliations:** Birkbeck, University of London, Reader in History of Architecture, London, UK

**Keywords:** lunatic asylums, seclusion, non-restraint movement, Lincoln Asylum, Hanwell Asylum, John Conolly, Robert Gardiner Hill

## Abstract

This article shows how the practice of seclusion—the confinement of asylum patients in locked rooms alone—entered the spotlight during the bitter controversy over the abolition of mechanical restraints in the late 1830s and early 1840s. Drawing on letters to *The Lancet*, asylum reports, reports of the Commissioners in Lunacy and polemical pamphlets, and focusing on the two asylums at the centre of the controversy, Lincoln and Hanwell, the article sets out the range of positions taken, from pro-restraint and anti-seclusion to anti-restraint and pro-seclusion. It shows how seclusion was associated with a lack of transparency, how it was seen as parallel to the disputed practice of solitary confinement in the prison system and how both the practice of seclusion and the single room itself were modified in the face of these challenges. John Conolly emerges as the most committed proponent of seclusion.

In the first three decades of the nineteenth century, mechanical restraint (the use of strait-jackets, chains, straps, muffs, sleeves and coercion chairs) was widely seen as a legitimate method of controlling and even calming violent patients in public lunatic asylums in Britain. The forcible confinement of violent patients in locked rooms alone (normally referred to as seclusion) was also accepted practice. The campaign beginning within asylum medicine in the late 1830s to abolish the devices and practices of mechanical restraint (known as the non-restraint movement) was for a small minority the first step towards the abolition of seclusion as well—this small minority recast both practices as abuses. More commonly, the introduction of non-restraint policies in asylums led to the *increased* use of seclusion, which was justified as an alternative to mechanical restraint—while strait-jackets and coercion chairs became instruments of harm, periods of forcible confinement in solitude were promoted as calming and restorative. A third group, asylum doctors who *opposed* the total abolition of mechanical restraint, were keen to exploit this seeming contradiction, using reported instances of forcible seclusion to discredit non-restraint’s promoters. The introduction of non-restraint has long been recognised as one of the pivotal moments in the history of psychiatry, and there is a rich literature devoted to it. This article turns the focus for the first time on seclusion as a bone of contention in the bitter debates about non-restraint in the late 1830s and early 1840s, asking why it was so deeply controversial and so multifarious in its associations.

Multiple pamphlets, lectures, reports, letters and editorials from the years when this debate was new, and most active—the late 1830s and early 1840s—allow access to its twists and turns, and to arguments rich with allusions to past psychiatric practices as well as to contemporary life beyond the asylum’s walls. The fact that the early years of non-restraint played out in two main institutions, the Lincoln Lunatic Asylum and the Middlesex County Lunatic Asylum at Hanwell, mean that these sources return again and again to particular lived instances within the spaces of the asylum—instances of the application and removal of restraint and cases in which seclusion is imposed or abstained from. Theory and practice are difficult to separate, and deliberately so, since all the antagonists in this battle insisted that their positions were firmly based on experience in the wards, and drew liberally (if of course selectively) on medical notes and case reports, excerpts from which filled the texts of this time.

The question of whether or not seclusion constituted harm was bound up with the anxiety that the asylum was betraying its inheritance as an enlightened institution. Locking people up behind closed doors was difficult to reconcile with efforts to make the asylum more transparent, to open it up to inspection from the outside. This kind of confinement also threatened to blur the all-important boundary between the prison and the asylum; repeated invocations of ‘solitary confinement’ by opponents of seclusion were meant, as will be shown, to summon the spectre of the prison in general and of highly controversial current systems of running prisons in particular. The disputants were preoccupied, as well, with the ways in which the spatial unit within which seclusion took place, normally the single bedroom, dampened, or intensified, the stimulation of the senses, both those of the patient in the room, and those of people in adjacent spaces. Whether seclusion was embraced or rejected, the scrutiny it underwent during this time of fundamental change in asylum management changed the spaces of the asylum; those epitomes of the carceral institution—inspection holes in doors and padded cells—became standard components as a result of non-restraint.

As Victorian residential institutions receive renewed attention from scholars, notably in the work of Jane Hamlett and her colleagues, there is also a renewed interest in the single unit—the spaces created for solitude within structures for collective living.^1^ Hamlett’s examination of private alcoves and single bedrooms in boarding schools and asylums draws on an innovative study by Thomas Crook looking at the phenomenon of the cubicle, especially as constructed in Victorian bathhouses and public conveniences.^2^ Both Crook and Hamlett are interested in these separate spaces as sites of privacy, havens for individuality and potential opportunities for deviance within the controlling and collective frameworks of the institution. Their view of the collective institution or public space as replete with solitary nooks and pockets of opportunity for individual expression offers a powerful counterpoint both to Erving Goffman’s total institution, with its regimented collective spaces and to Michel Foucault’s Benthamite rings of back-lit cells offering up the body of the inmate for surveillance.

What can be seen in the debate, examined in this article, about the form the asylum should take under the pressure of the non-restraint imperative is that the role of the single space for solitude was crucial and also that it could be envisioned as a space of privacy and potential deviance as well as one of control and normalisation, depending on the position taken in the dispute—the whole range of associations were available to and deployed by the doctors, officials and interested commentators who made their voices heard. What also emerges though is the power and significance of the prison as a counter-example. Indeed it was precisely because of the seeming similarity between asylums and prisons that asylum designers were so keen to differentiate them, and to embrace what Michael Donnelly called ‘a special “ethos” of confinement’, to be seen to confine differently, indeed to mask the fact of confinement as much as possible. According to Donnelly, the new asylum designs emerging from the lunacy reforms of the 1840s ‘conscientiously severed the older associations of asylums with the apparatus of prisons (and their gloomy, barred spaces, their clanking chains, their stench, their rude keepers …).’^3^ Attention to the debates about seclusion show that there was an equally strong impulse to differentiate asylums from *contemporary* prison design, and particularly from the separate system prison exemplified by the new National Penitentiary at Pentonville, then being planned. As Michael Ignatieff has shown in his study of British carceral policy, the battles over the separate system were not concerned with stench or the clanking of chains—but with the hugely fraught question of whether solitary confinement was a force for good or for ill.^4^

## Battles over Restraint and the Role of Seclusion

The non-restraint movement began in bitter controversy. A physician and radical political activist, Edward Parker Charlesworth, and his young protégé and house surgeon at the small charitably-funded Lincoln Lunatic Asylum, Robert Gardiner Hill, threw down the gauntlet when Charlesworth arranged for Hill to deliver a lecture to the Lincoln Mechanics’ Institute in June 1838, which was published in 1839 under the title *The Total Abolition of Personal Restraint in the Treatment of the Insane*.^5^ John Conolly, superintendant of the much larger and more nationally prominent Hanwell Asylum to the west of London, reviewed Hill’s pamphlet positively, and announced in his reports to the public body overseeing his work (the magistrates of the county of Middlesex), his intention of introducing the non-restraint system (as it had come to be called) at Hanwell.^6^ Non-restraint, as Hill defined it, was a mode of asylum management which rigorously abstained from any form of ‘mechanical restraint’ of patients, no matter how violent their behaviour was. There was some ambiguity in Hill’s definition of ‘personal’, or ‘mechanical’ restraint (which he elsewhere referred to as ‘severity’), but in the ensuing commentary it was understood to mean the confinement of a person’s limbs by means of a physical device or devices.^7^ (The most common devices were chairs to which patients were strapped, leather straps attaching patients to bedsteads, strait-jackets preventing the movement of the arms and muffs and hobbles restricting the movement of hands or feet.)^8^ Hill had instituted non-restraint at the Lincoln Asylum for the previous 16 months, and claimed excellent results.^9^ He therefore advocated its introduction in all asylums.

Hill’s challenge to the emergent profession of asylum-based psychiatry was influentially supported by Conolly, but also inspired reactions ranging from scepticism to outright hostility. *The Lancet*, whose editor, Thomas Wakley, was always on the look out for controversy, reprinted a passage from the annual report of the West Riding Asylum at Wakefield in which the superintendent, C. C. Corsellis, expressed doubts about the advisability of abolishing restraints; Wakley welcomed readers’ views.^10^ Hill wrote with a strongly worded defence of his system, but he had opponents within his own institution in the form of Samuel Hadwen and William Cookson, who themselves felt embattled and wrote to *The Lancet*, triggering further rebuttals from Hill, who was supported by the editor.^11^ Regular letters continued throughout 1840 and into 1841, and from 1840 to 1844, Conolly included extensive discussions of seclusion and non-restraint in his annual reports on Hanwell. Meanwhile, the non-restraint debate was taken up by the inspecting body known as the Metropolitan Commissioners in Lunacy in their report of 1844. It is this range of texts from the first six years of non-restraint that are my main source material. They come from a period of time and a range of sources that are of central importance to the history of psychiatry in Britain and indeed beyond. Lincoln was a small local asylum with a middle class clientele—all of which made experimentation possible. The radical rejection of conventional wisdom, that took place there combined with Charlesworth’s talent for public relations, meant that Lincoln asylum’s example loomed larger than seemed warranted by its significance on the national scene. Hanwell on the other hand, could not have been a more prominent stage for these reforms and controversies, because of its prominence as one of the largest of the country asylums, and because it served the capital. The non-restraint debate was a turning point too for the Commissioners in Lunacy, the point at which they extended their reach nationally and consolidated their position around the promotion of non-restraint.^12^ Their ambivalence towards seclusion was therefore highly significant.

The issue of the forcible confinement of patients in a room alone (seclusion) was not Robert Gardiner Hill’s main concern, and did not fall within the forms of restraint subject to his ‘total abolition’ proposal. Hill’s opponents, by contrast, were keen to talk about seclusion, and especially about instances of its use under a non-restraint regime. They sought to defuse the power of the ‘non-restraint’ slogan by sowing confusion about what restraint meant, and who could be accused of it. John Adams, a Middlesex magistrate and prominent supporter of both Hill and Conolly, wrote to *The Lancet*, recognising that one of Hill’s opponents was playing with shifting definitions:

The word *restraint* here is very equivocal, for I know it is applied by the supporters of the restraint system to the confinement in a separate apartment, as well as to *muscular*, or, in other words, *instrumental restraints*; such, for example, as strait-waistcoats, sleeves, handcuffs, &c., although the supporters of the non-restraint system expressly limit themselves to the latter definition, and consider *seclusion* and quiet, in many cases, as great auxiliaries to the cure.^13^

To accuse the non-restraint humanitarians of locking patients up alone could only be effective if that practice could be made out to be as objectionable as (or even worse than) the physical restraint of patients with the armoury of medieval-seeming devices to which Hill and others objected. William Cookson, one of Charlesworths’ rival physicians at Lincoln, declared himself in a letter to *The Lancet*

to be a most determined opponent of the system which has been pursued in the Lincoln Asylum; a system which I do not admit to be a system of non-restraint. I believe that the abolition of visible restraint in the Lincoln Asylum has been followed by much secret oppression, much hidden violence, and by many revolting practices, a thousand times more dangerous than mechanical restraint, because they can be neither so easily detected, nor so readily controlled.^14^

Cookson’s primary example was the long-term seclusion of a Lincoln asylum patient known as Miss A. In his letter, as well as in a letter from Cookson’s ally Samuel Hadwen, Hill’s predecessor as house surgeon at the Lincoln asylum (who was now a governor), the case of Miss A, described as an extremely violent and unmanageable patient in the female refractory ward, is prime evidence. Previously, Miss A had been restrained from harming herself and others during violent episodes by the application of mechanical restraint devices, both while inside her room and sometimes also outside, in the galleries and day rooms. Indeed her case, according to Hadwen, was one ‘the judicious and humane management of which, I fearlessly assert, can never be accomplished, by any means at present known, without recourse to instrumental restraint’.^15^ After Hill’s abolition of all restraint devices, Cookson and Hadwen claimed, the only option had been to confine Miss A to her room. Hadwen presents this solution as an imprisonment and a damning injunction against the then new system:

Here is a difficult, and, under the present system, a wholly unmanageable case of insanity; the unhappy victim of which spends day after day, and night after night, in solitary confinement, in a small, ill-ventilated, ill-lighted, and oppressive apartment; a restraint known to be the most insupportable, and from which the most hardened criminals shrink with dread abhorrence, and, with its additional accompaniments in this instance, a species of restraint infinitely more calculated to convert the sane man into a lunatic, than to restore the lunatic to health. Secluded from the free light of heaven, from the pure air, the bracing exercise essential to health, what chance has this most unfortunate person of obtaining the deliverance from the calamity that made her an inmate of the asylum? An asylum? Alas! It is to her a dreadful dungeon! … I am perfectly horrified that such a reprehensible mode of treatment should be persisted in by those who are continually publishing to the world the superiority of the *management* of the insane at Lincoln.^16^

Hill objected in a subsequent letter that when Hadwen was in charge, he himself ‘used seclusion very freely, though he affects so much compassion for the feelings of a patient who has been subjected to it’.^17^ Indeed several instances and arguments used by the opponents of non-restraint do not constitute objections to seclusion per se (although they sit alongside passionate denunciations of the practice), but rather to secluding a patient without also additionally restraining her (by, for example, fastening her to the bedstead). It is the patient at liberty within the locked room that leads to situations that are criticised by the opponents of non-restraint both within Lincoln and beyond. Hadwen and Cookson detail the degeneration of Miss A’s room into a foul-smelling cess-pit, covered in the remnants of her meals as well as in urine and faeces. Cookson reproduced an extract from the physician’s log recording a day in which ‘Miss A. could not be removed from her room on account of her extreme violence. … She throws herself down with violence on the floor; and then when anyone comes near her door, she starts up and beats it with her fists’.^18^ Cookson recounts a decision made to bolt a chamber pot to the floor of Miss A’s room, since she had been using the standard pot provided as a missile. For Cookson, such a dire recourse begs the question: ‘When the proprietors of lunatic asylums were libelled as people who chained up lunatics from sordid or unworthy considerations, why was it not stated that in the very house from which the ungenerous words were addressed, it had been judged necessary, as a matter of safety, to convert a patient’s bed-room into an *oubliette*?’^19^ The Wakefield superintendent Corsellis entered the fray with his own letters to *The Lancet.* He saw the room of seclusion as a place in which the patient should lie down and rest, rather than roaming around—if he cannot be persuaded to do this, the only solution, where restraints (he suggests ‘a pair of ticking sleeves, an article so contrived that it can neither hurt him nor be fastened too tightly, and … a narrow strap of leather communicating with the staple in the bedstead, to prevent him from getting out’) are not permitted, is a brutal physical battle involving two keepers holding him down in the bed.^20^

The early 1840s also saw the expansion of the duties of the Metropolitan Commissioners in Lunacy, who were charged by Parliament in 1842 with inspecting and reporting on conditions in public and private institutions in which the insane were housed and treated, not only in the Metropolitan area but across England.^21^ In the resulting report, published in 1844, the non-restraint movement was a central concern. At this stage, the Commissioners adopted a moderate position, embracing the general tendency towards mildness and liberality in institutions, while allowing for a remaining role for some forms of restraint.^22^ The report took up the issue of restraint’s definition and the role of seclusion:

Those who profess the entire disuse of restraint, employ manual force and seclusion as parts of their method of management, maintaining that such measures are consistent with a system of non-restraint. It is said by these persons that when any of the limbs (as the legs or the hands of a patient) are confined by the strait-jacket, the belt, or by straps or gloves, he is under restraint. But in cases where he is held by the hands of attendants, or when he is for any excitement or violence forced by manual strength into a small chamber or cell, and left there, it is said that restraint is not employed, and the method adopted in these cases, is called ‘the non-restraint system’.

The report went on:

Here restraint of some form or other is manifest; and even in those cases where the patient is forced into a cell by manual strength, and prevented from leaving it until his fit of excitement shall have passed, it is difficult to understand how this also can be reconciled with the profession of abstaining from all restraint whatsoever, so as to be correctly termed ‘Non-restraint’. It seems to us that these measures are only particular modes of restraint, the relative advantages of which must depend altogether on the results.^23^

The tone here is more measured than what we read in the *Lancet* letters, but what remains is a fairly fundamental scepticism about non-restraint as a pure ideological position—and the pinpointing of the promotion of seclusion as non-restraint’s weak point. Whereas in 1840 the focus was on Hill and practices at Lincoln, by 1844 it had moved to Conolly and his extremely influential introduction of non-restraint at the much larger and more prominently located Hanwell asylum. The Commissioners, knowing the readers of its report on the nation’s asylums would be particularly interested in its account of the Hanwell showcase for non-restraint, expressed themselves in distinctly ambivalent terms:

The system of non-restraint at Hanwell has been carried on by mild and kind treatment, by an increase in the numbers of attendants, and by adopting seclusion or solitary confinement, sometimes in darkened cells, in lieu of mechanical restraint. At our visit to this Asylum in 1843, there was no patient under mechanical restraint; but we saw a violent female lunatic, who had been endeavouring to bite other persons as well as herself, seized by four or five of the nurses, and after a violent and protracted struggle, forced with great difficulty into and fastened in, one of the cells. During this scene, there was much confusion in the ward, and the great efforts of the patient to liberate herself, and (after her seclusion) the violence with which she struck the door of the cell, and threw herself against it, must have greatly exhausted her.^24^

The Commissioners stressed the role (and what they presented as the reality) of seclusion in order to problematise non-restraint, pointing to its limits as an ideological position. They also focused attention on an aspect of seclusion that had not featured in the earlier criticisms. While Hadwen, Cookson and Corsellis evoked dire situations inside the locked room, the Commissioners bore witness to the process by which the unwilling patient was forced into the room in the first place, involving violence and coercion.

Conolly himself was indeed an enthusiastic advocate (and practitioner) of the forcible seclusion of patients during spells of violence and threats to themselves and others, presenting it as one of the most important alternatives to mechanical restraint in such cases.^25^ At the same time, he recognised clearly the symbolic challenge this practice posed to the non-restraint project, which was supposed to be characterised by humanity, kindness and justice. At the end of 1840, having observed the controversy sparked by Hill’s introduction of non-restraint at Lincoln Asylum and the fate of Miss A in *The Lancet*, Conolly composed a point by point defence of seclusion in his annual report to the Middlesex Magistrates who had oversight of the asylum:

All the substitutes for restraint are, like restraint itself, liable to be abused; but none can be made such instruments of cruelty by abuse. All are also liable to great misrepresentation: and none more so than that which is of all the most useful, the most simple, and the most approved of by the highest medical authorities—namely, seclusion.^26^

In place of the misrepresentations he deplored, Conolly offers a definition of seclusion that, while not denying that the practice consists of locking a person in a small room, downplays entirely its coercive and carceral aspects. Instead he puts seclusion on a medico-scientific plane, and claims for it a well-founded therapeutic basis:

By seclusion is meant temporary protection of the maniac from the ordinary stimuli acting upon the senses in the refractory wards of a lunatic asylum. He is abstracted from noise, from the spectacle of a crowd of lunatics, from meeting those who are almost as violent as himself, and from every object likely to add to his irritation.^27^

He then immediately goes on to admit that seclusion has other, less benign, associations, pinpointing the ‘mode in which seclusion is effected’ as the factor which can tip it away from cure and towards punishment and/or neglect: ‘If resorted to with violence, if accompanied with expressions of anger or contempt, if stigmatised as a punishment, and if followed by neglect, it may produce all the evil moral effects of restraint itself.’^28^ Conolly developed a series of responses to each of the potential pitfalls of seclusion, some of which were in the form of strict guidelines for attendants. He gave a detailed account, for instance, of precisely how attendants should get the patient into the seclusion room—and how they should exercise emotional control over themselves as well as over the patient when doing so:

Three or four attendants, possessed of courage and a good temper, should surround him; and telling him that he would be much better if quiet, and in his own room, should endeavour, by gentle occasional efforts, to induce him to walk into it. It will sometimes be found, that although he protests loudly against the measure, his steps gradually proceed in the direction required. At the same time, steadiness and strength may be required to prevent him retrograding; but well-qualified attendants will not, on this account, resort to violence. If he strikes and kicks them, they must, of course effect their purpose as speedily as possible, and with steadiness, and even with force; but always without passion.^29^

Other responses to seclusion’s potential problems took the form of adjustments to the built fabric of the asylum, softening the cell, and making its interior visible from beyond the locked door—these are discussed in more detail below.

Back in Lincoln in the early 1840s, a third position was developing. While the opponents of non-restraint attacked the use of seclusion by non-restraint proponents as hypocrisy and abuse, and Conolly embraced seclusion as a therapeutic tool of great use to non-restraint practitioners, Hill and his allies moved towards the abolition of both restraint *and* seclusion. Hill had in fact classified seclusion as a form of restraint in his 1838 lecture, but conceded that it could be used in cases where the conditions for total abolition of restraint did not exist (that is, where the asylum building was not yet well adapted for full non-restraint and there was a shortage of sufficient attendants).^30^

It was the case of Miss A, her long-term seclusion under Hill’s successor as house surgeon, William Smith, and perhaps above all the way in which her treatment was used so publicly by Hill’s opponents to discredit his campaign, that led to seclusion itself being abolished at Lincoln. According to a letter to the asylum governors dated October 1841, Smith had decided henceforth to treat C.A. (as he referred to her) without recourse to seclusion. The success of his ‘experiment’, he wrote, ‘impressed me with a conviction that solitary confinement, as a means of control, may be successfully and usefully dispensed with in this Institution, under well disposed and practised attendants and vigilant superintendence, as instrumental restraint has already been’. He predicted that he would have to be more vigilant than usual towards the attendants, who had ‘become accustomed to rely upon seclusion, instead of increased attention, in troublesome cases’. He also advocated a degree of tolerance of ‘lunatic violence, under sudden impulse’ which, he wrote, ‘must be expected in Lunatic Asylums, and can never be totally suppressed, except by perpetual restraint, or perpetual seclusion, far more injurious and distressing than an occasional blow under temporary excitement’. In any case, he pointed out, seclusion did not in fact prevent violent episodes: ‘the official books exhibit evidence weekly of violent collisions, during the long period of C.A.’s seclusion, and the general employment of this agent, proving its inefficacy as a source of protection’.^31^

Hill, who was at this point Chairman of the Asylum’s Board of Governors, announced in the 1842 annual report that Smith’s successful experiment and subsequent realisation that seclusion was unnecessary ‘even under the most peculiar cases’ had led to a change in official asylum policy, so that ‘the Solitary Confinement termed the Seclusion of the Insane, now no longer exists in this Institution as a means of control’.^32^ There were hopes that Lincoln’s example would again, as it had a few years earlier with non-restraint, lead to widespread change. Smith, in a second letter dated January 1842, announced the success of his ‘experiment of abolishing altogether solitary confinement in this Institution’ and went further: ‘the extraordinary improvement which has followed in the good order of the North Galleries, remarked upon by both the official visitors and by strangers, confirms my belief that this practice may be safely and … advantageously introduced into other Asylums, as an accompaniment and part of the humane system of the disuse of instruments …’^33^ Lincoln thus set itself up as a counter model to Hanwell (as the non-restraint non-seclusion examplar, versus the non-restraint pro-seclusion one), but Hanwell’s influence was much more powerful. The Commissioners noted in their 1844 report that ‘Lincoln Asylum is the only place in which even seclusion is not resorted to’.^34^

The forcible seclusion of patients, then, led to highly distinct reactions, even among those, such as Conolly and Hill, who had identical views on the need to refrain from any use of mechanical restraints. Behind the various stances on seclusion were a set of powerful associations conjured up by the phenomenon, or image, of a person locked in a room alone. The lunacy reform impulse of this period put great weight on transparency, and the discovery by inspectors of forgotten and fettered wretches behind the bolted doors of cramped cells was a recurrent trope that coloured attitudes to seclusion as a practice within the reformed asylum. Solitude in a private room as a balm for the active mind was embraced in the domestic built environment generally, but enforced solitude was more readily associated with solitary confinement as it was being instituted at that time in the reformed prison system. And both supporters and opponents of seclusion saw the single room in terms of what it could do to sensory perception—dampening it and blocking it out, or condensing and intensifying it.

## Seclusion and Transparency

Small rooms intended for one person—the standard term for them was cells—had formed the basic spatial unit of the asylum for centuries, and although the existence of cells or single rooms did not necessarily lead to patients’ forcible confinement in them, the associations between cells, isolation, unhygienic conditions and locked doors were frequent.^35^ Hill, in a historical preamble to *Total Abolition*, refers to early asylums as collections of oppressive and inhumane individual spatial units: ‘Their rooms or cells were uniformly loathsome from dirt; and in many places on the Continent, Lunatics were confined in cages, through the bars of which food and straw were thrust in to them, and they were daily exhibited to visitors, who paid a certain sum to see them, as is done with wild beasts.’^36^ The non-restraint movement took place against the background of a period of lunacy reform in England which put increasing emphasis on the role of objective inspectors and lay visitors (of a different sort from the prurient paying visitors of the past), making unannounced visits to private and public asylums and looking behind every locked door for evidence of inhumane conditions and abuse. The unveiling of abuses was dramatised by accounts of the discovery of chained patients, whose suffering (and above all their invisibility) was exacerbated by their confinement to a dark and filthy cell, where they had languished invisible and forgotten, for months or years.^37^ Hill was eager to show how the Lincoln Asylum met the highest standards of transparency and included in the appendices to *Total Abolition* multiple extracts from asylum reports and logs detailing measures taken to ensure that practices, and spaces, were open, visible and acknowledged. On an administrative level, there was the establishment of a register recording the frequency and length of the use of restraints (one of which, listed alongside ‘hobbles’, ‘the chair’ and ‘the strait-waistcoat’, was the ‘noisy cell’), and a book, on open display in the entrance hall, in which visitors could record any abuses witnessed.^38^ All the other measures he pointed to concerned the building itself, including the Board’s resolution ‘that a Plan of the Asylum be … hung up [in the entrance hall], to enable [visitors] to ascertain whether any part of the building has been concealed from inspection’.^39^ In 1835, the doors opening into and dividing different parts of the galleries were partly glazed in another transparency measure, since ‘the opportunities of neglect and harshness behind close and closed doors amidst incompetent witnesses, must be so unlimited, that every obstruction of observation may be considered as an exposure of these institutions to the risk of such consequences’.^40^ In this context, the claim by Hill’s opponent, Cookson, that the non-restraint system at Lincoln had led to ‘much secret oppression, much hidden violence … a thousand times more dangerous than mechanical restraint, because they [cannot] be so easily detected’ effectively targeted seclusion as the Achilles heel of the transparent, non-restraint institution.

The Commissioners in their 1844 report also worried that because seclusion was ‘less visible’ than mechanical restraint, it was ‘more liable to abuse, and less capable of detection’.^41^ One remedy they proposed was the introduction of a requirement that all asylums maintain a register of every instance of seclusion, on the same basis as a restraint register, which was already required. A new Lunatics Act in 1845, introduced in response to the Commissioners’ report, formalised the requirement that all institutions for the treatment of the insane keep a register of both restraint and seclusion.^42^ Conolly came to agree that periods of seclusion should be recorded, writing in 1856 that a register helped to secure ‘all the advantages of seclusion, without any abuse of it’.^43^

But Conolly initially responded to the concerns about the invisibility of seclusion by promoting ways of ameliorating, by means of spatial configuration and design, the potential of locked single rooms to hide patients from both care and inspection. Seclusion should happen, not in a separate wing or tract removed from the rest of the institution, but in ‘a quiet bed-room opening directly out of the gallery’.^44^ When ‘troublesome patients were securely fastened down’ within their rooms, they were neglected, and ‘nobody seemed to care what condition they were in’.^45^ Seclusion without mechanical restraint required and resulted in more attention from attendants and doctors, although the importance of keeping the patient protected from stimulation meant that opening the door and looking in, or entering, would be counterproductive and possibly dangerous. Conolly therefore advocated the use of what he called ‘inspection plates’, covered openings in the door allowing the attendant in the gallery to see all parts of each single room: ‘By occasionally looking through the inspection-plate, the attendant is enabled to ascertain the effect of the seclusion; and the medical officers, to whom every seclusion is, or ought to be, immediately reported, are enabled to judge of the propriety of continuing or putting an end to it’.^46^ Here too was the solution to the locked room as an impediment to inspection by outsider observers: ‘In conducting visitors through the asylum, their attention is generally directed to the cases actually in seclusion … whom they are commonly able to observe without occasioning them any disturbance’.^47^ At Lincoln, seclusion was seen to be incompatible with the transparent, liberal institution, and was abolished. At Hanwell, by contrast, a framework of bureaucracy and design was developed which was intended to compensate for seclusion’s transgressions against transparency.

## Seclusion and Solitary Confinement

According to Conolly, patients in the midst of a violent spell, once secluded and thus removed from the ‘stimuli’ of the refractory wards, would not only calm down, but would engage in distinctly genteel activities:

The patient who was five minutes before filling the gallery or the air with shouts, and exhausting himself in vehement and menacing actions, is found at once to cease to shout and threaten; to walk up and down his room, quickly at first, but soon more quietly; then to sit down and read, or to lie down and sleep. Women so secluded will walk about for a short time, and then take up a needle and begin to sew.^48^

The notion of time spent in a room alone as both calming and civilising chimed with idyllic visions of privacy within the home, which were a feature of Victorian thinking about domestic space.^49^ Indeed, Conolly’s preferred term, ‘seclusion’, had associations with romantic, freely-chosen solitude.^50^

Conolly strongly objected to another term frequently used in an asylum context for locking a patient up in solitude: ‘solitary confinement’. Samuel Hadwen seems to have been the first participant in the battle over non-restraint to use the term. In his letter to *The Lancet* (quoted above), attacking Hill and the abolition of restraints at the Lincoln Asylum, he described the patient Miss A spending ‘day after day, and night after night, in solitary confinement’. Solitary confinement was not only ‘a restraint’, he wrote, but one ‘from which the most hardened criminals shrink with dread abhorrence’. Not only that, but it is ‘infinitely more calculated to convert the sane man into a lunatic, than to restore the lunatic to health’.^51^ For *The Lancet*’s readers, Hadwen’s choice of terms and associations would have immediately called to mind the ongoing project of prison reform in the Anglo-American world, and specifically controversies over modes of prison design and management in which all prisoners spent both night and day in their cells in solitary confinement. Indeed just a few months earlier, another correspondent to *The Lancet* had addressed specifically the impact of such a system on prisoners’ mental health. William Simpson wrote, commenting on the plans for the model penitentiary at Pentonville, which had then just commenced construction:

As I am led to understand that the Government are about to make an experiment upon the solitary confinement … system of punishment, in the model prison, which is now being erected, I beg to call your attention to the subject, lest it should be adopted and carried too far, without looking into the effects produced by the same system in other countries where it has been tried for some time, particularly in America and Belgium. From reports published in America we find, that long solitary confinement has the effect of debilitating the mind, as well as the body, and after their time has expired, many criminals are thrown upon the world in a state of complete *idiotcy, besides having contracted habits contrary to nature and prejudicial to health.*^52^

As Michael Ignatieff has shown, British observers need not have looked to America or Belgium for reasons to have misgivings about the use of solitary confinement in prisons. The practice was home-grown, first being put into practice when George Onesiphorous Paul in Gloucestershire and justices in Berkshire and Sussex introduced extreme forms of solitary confinement in the late eighteenth century, confining prisoners in solitary cells for all but two–three hours a day, and even building separate exercise pens in their reformed prisons, refered to as ‘penitentiaries’. These reformers took John Howard’s principles of separation (developed in his influential accounts of visits to prisons across Europe and based on the dangers of association between criminals) to an extreme. Howard himself disapproved, fearing ‘that unbroken solitude would break the spirit of inmates and lead them into either “insensibility or despair”’.^53^ As early as the 1790s, there was a concerted campaign against the practice, initiated by political prisoners subjected to solitary confinement regimes and taken up by Sir Francis Burdett, who associated penitentiaries with Bastilles and suceeded in moving public and progressive political opinion against solitary confinement, which came to be associated with cruelty.^54^ So by the time the National Penitentiary at Pentonville was under construction, solitary confinement was a subject of vivid contention.

Conolly objected in Hanwell’s annual report of 1841 to ‘the extravagant notions of *seclusion* set forth by opponents of the non-restraint system’, and specifically to the idea that seclusion was ‘an imprisonment, productive of every moral and physical evil’.^55^ But Hill and his protégé Smith, at Lincoln, once they had decided to abolish seclusion, as well as restraint, adopted their adversaries’ terminology: ‘The Solitary Confinement termed the Seclusion of the Insane, now no longer exists in this Institution as a means of control’.^56^ In 1844, the term was used again in the Lunacy Commissioners’ report as a synonym for seclusion, the Commissioners also alluding to current concerns about solitary confinement’s impact on mental and physical health:

As solitary confinement is coming into more general use, as a remedy in Asylums, and as persons who have been subjected to its operations for long periods, have become insane, we feel that we ought to notice the practice so far as it may be employed in the treatment of lunatics. As a temporary remedy, for very short periods, in cases of paroxysms and of high excitement, we believe seclusion to be a valuable remedy. We are convinced, however, that it ought to be used only for short periods, and that it should not be permitted as a means of managing and treating those persons who are permanently violent and dangerous. Long solitary confinement of any person in a cell is calculated to destroy his bodily health.^57^

Hadwen had claimed that Miss A’s experience of extended confinement in her room at Lincoln had subverted the very notion of the institution as an asylum from the indignities suffered by the mentally ill in the world outside: ‘An asylum? Alas! It is to her a dreadful dungeon!’^58^ The insistence by opponents of non-restraint that seclusion was in fact solitary confinement blurred the distinction between asylum and prison—a distinction particularly important for the supporters of non-restraint and of asylum reform in general.

## Seclusion and the Senses

Conolly made another distinction between seclusion and solitary confinement. While seclusion was ‘a simple exclusion of irritations from an irritable mind’, solitary confinement constituted ‘a privation of almost all the stimuli upon which the integrity of intellectual and physical life depends’.^59^ The locked single room was recognised as a powerful tool for the control and manipulation of stimuli (to use Conolly’s term)—the occupant’s range and intensity of sensory perception could be dampened or strictly curtailed when they were secluded. As Conolly acknowledged when he distinguished between seclusion and solitary confinement, the power to control sensory input could be deployed therapeutically—or punitively. We see this issue at play in the repeated references to levels of light in the cell or single room. Campaigners against mechanical restraint often evoked the ‘dark cell’ as the site of hidden abuses. In one of his letters to *The Lancet*, Robert Gardiner Hill envisioned a situation in which a patient, having insulted a keeper, is violently restrained, forced into a strait-jacket and hobbles, and ‘to sum all up … he is chained to a wall in a small dark room, and the door is closed upon him’.^60^

In the same months in which it published the correspondence about Hill’s innovations at Lincoln, *The Lancet* also published allegations of abuses at the ancient asylum of Bethlem in London. Two reformers (Charles Tulk and John Adams, magistrates closely involved in the introduction of non-restraint at Hanwell) visited Bethlem and were informed by a patient that he had been subjected to weeks of ‘solitary confinement in a dark cell’.^61^ Being on site, they decided to give themselves a direct taste of the experience:

We went into the cell, and had the door shut upon us, and I could not perceive the faintest glimmering of light. It was not a darkened cell, in which the light is softened, not excluded, but truly a dark cell, in which I could perceive no crevice, even around the door, by which light could enter; while a window shutter, near the top of the cell, was closed and locked, so as effectually to shut it out on the side opposite the door. Here [the patient] had been confined.

Tulk referred to this ‘solitary confinement in utter darkness’ as ‘a punishment more severe by far than any proposed separate confinement for prisoners’.^62^ The severity of the suffering was compounded by the real and symbolic power of darkness, combined with enclosure, to conceal abuses from the public eye.

The distinction Tulk makes between ‘a darkened cell, in which the light is softened, not excluded’ and something that was ‘truly a dark cell’ is interesting. Leonard Smith points out that by the time Conolly began advocating it as an alternative to mechanical restraint, the isolation of agitated patients in a darkened room had been established practice in asylums for decades—justified by the therapeutic desire to limit external stimulation.^63^ The report of the parliamentary Select Committee on Lunatic Asylums in 1827 included a series of questions for inspectors to put to asylum superintendants. One was: ‘Are dark solitary rooms made use of with advantage in cases of violent maniacal paroxysms?’^64^ The rationale was presumably that by being placed in conditions, even during the daytime, that simulated night and thus facilitated rest and sleep, the patient could be calmed.^65^

Utter darkness, though, was the medium of fear, privation and concealment, and Conolly emphasised that he advocated a partial, rather than total, restriction of the entry of light into the room. A violent patient could not be trusted alone in a room with an unprotected window, so the window needed to be furnished with an interior shutter and lock. But, wrote Conolly, ‘the room is not always darkened even by the closure of the shutter, and it is never completely dark’.^66^ Letting some light in was important for inspection: ‘Sufficient light should be admitted through holes made in the window-shutter to enable the attendants, by looking through the inspection-plate in the door, frequently to ascertain the state of the patient.’^67^ Light and relative darkness could be prescribed like medicine; Conolly ordered seclusion ‘sometimes with the light partially excluded, sometimes almost entirely’, but ‘seclusion in total darkness is seldom, or perhaps never, necessary; and it would often be a dreadful punishment, either much aggravating the patient’s agitation, or exciting frightful thoughts’.^68^ The point in any case was to achieve ‘repose of the brain’, and as we have seen Conolly’s ideal seclusion resulted in the patient sitting or lying down, sewing, reading or sleeping.

Conolly thought of the single room not only as a screen or shade, but also as a muffler. Silence was another of Conolly’s aims for the agitated patient, who, once secluded, was ‘abstracted from noise’.^69^ The Commissioners reported a markedly different aural experience of seclusion under Conolly’s direction at Hanwell, noting ‘the violence with which [a secluded patient] struck the door of the cell, and threw herself against it’.^70^ Miss A, at Lincoln, was reported (by Cookson and Hadwen, assembling evidence against the results, as they saw it, of Hill’s non-restraint system) to have not only thrown herself on the floor and beat on the door, but also ‘bellow[ed] forth the most horrid blasphemies and threats at some imaginary beings’.^71^ She threw her food on the floor, urinated and defecated (and was therefore ‘compelled to inhale an atmosphere loaded with exhalations from her own urine and faeces’) and spent the hours of 10 pm to 3 am ‘knocking her head and limbs against the door’ with the result that ‘the door and boards of [her] cell are frequently stained with blood’.^72^

The room is no longer here a tool for the control, from without, of the ways in which sensory stimuli might act on the patient. Instead, the opponents (or, in the case of the Commissioners, the problematisers) of seclusion, point to how the room can be transformed into a kind of sensory condenser by the patient locked inside. A shouting voice that would be dissipated in a long corridor or in the airing court is focused and magnified. Urine and faeces, rather than being removed, fester in close proximity, assailing all the senses. And the patient’s compulsive and violent bodily contact with the walls, floor and door both stains the room and unleashes the most intense stimulus of all: pain. Those who saw seclusion as a scandal may have objected to it on an abstract level as imprisonment, but these accounts reveal a more visceral revulsion against the ways in which the combination of locked room and agitated patient condenses and magnifies the sensory experiences of both patient and inspecting doctor, experiences that might otherwise be dissipated or controlled.

What then did the various players in the debate imagine as the solution to the problem of an oppressive, or noisy, or painful seclusion? For opponents of non-restraint such as Corsellis, seclusion could be rendered more predictable and less destructive and unhygienic if it was supplemented by mechanical restraints: the patient would be more likely to sleep and rest, and would be unable to bang her head against the walls if she was strapped to the bed.^73^ The asylum doctors Bucknill and Tuke, looking back on this period, articulated a pro-restraint, anti-seclusion position that was not made explicit in the debates I have looked at, but may well have been implicit: ‘The character of seclusion, as a remedy, has never recovered from the attacks on it made by the advocates of mechanical restraint. They represented, truly enough, that a patient walking about pleasure-grounds, with his arms tied to his sides, was capable of more enjoyment than he would be if he were shut up in a dark and narrow cell, with all his limbs at liberty’.^74^

The position in support of seclusion from the non-restraint camp—that is, Conolly’s position—was that seclusion should always be well monitored and temporary, and of course always effected without resort to mechanical restraints. In addition, the room itself should be carefully designed to promote successful, calming, non-punitive seclusion. In most cases, Conolly believed that an orderly, well furnished patient bedroom, with additional security features such as a shutter on the inside of the window, a bed bolted to the floor and an inspection plate in the door for surveillance, would ensure quiet, safe and well-monitored seclusions.^75^

Conolly conceded, however, that there would be cases of patients for which this solution would not be sufficient. For these, he proposed what seems to have been at this point (when he was writing, in 1844) a novel, if not entirely new type of room, devised specifically for seclusion of extremely violent patients without recourse to mechanical restraints: ‘a room of which the floor is a bed, and the four walls are padded’.^76^ In a later publication from 1856, Conolly described the purpose and fitting out of a padded room in detail:

The great advantage of a padded room in all these cases, is that it renders both mechanical restraints and muscular force unnecessary for the control of even the most violent patients. Such an apartment, at Hanwell, is prepared by a thick soft padding of coir (cocoa-nut fibre), enclosed in ticken, fastened to wooden frames, and affixed to the four walls of the room—the padding extending from the floor to a height above the ordinary reach of a patient. The whole floor of the room is padded also, or covered with a thick mattress, of the same material as the padded walls, so that it makes a complete bed. In general, the room contains no furniture except bolsters or pillows, also covered with strong ticken. The window is guarded by a close wire-blind, which admits light and air, but prevents access on the part of the patient to the glass or window frames. … In a room so arranged the patient cannot easily injure himself, or receive accidental injury.^77^

According to the Commissioners in their 1844 report, ‘great numbers of the superintendents of public, and of the proprietors of private Asylums throughout the country are fitting up and bringing into use solitary cells, and padded rooms for violent and unmanageable Lunatics’.^78^

The padded room was a single room defanged, divested of sharp corners, and hard surfaces, furnishings and fittings. It was the counterform to the old cell in which the patient was chained so that he did not damage the room, or damage himself by way of the room. It was a space within the restricted confines of which the patient could move freely—the closest modern parallel would be the toddler’s playpen. While the seclusion of less violent patients could take place within an ordinary bedroom, the padded room was thoroughly institutional and anti-domestic: no reading or sewing would happen in here. It both softened and reinforced the boundaries between the space within and any spaces beyond, not only obviating any harm the patient might do to the asylum’s property and himself, but also muffling and muting the patient’s ability to make an impact on his wider surroundings, through bellowing and beating at the door.

While Hanwell was having padded rooms installed, the doctors at Lincoln countered the solitude and the mess of seclusion in a radically different way; instead of muffling the agitated patient’s energies, they proposed redirecting them via energetic and outward-facing activity. William Smith reported that his ‘experiment’, the treatment of Miss A without recourse to either restraint or seclusion, had, after ‘a few outbreaks’ rendered her ‘tractable, good-natured, sensible of kindness, [and] conscious of approbation’. And social: she ‘accompanies her attendant … on business with the shops in the town, and mixes harmlessly and happily with the other patients, at their monthly tea drinkings and dances’.^79^ Hill’s announcement that seclusion had been abolished led directly on (in the annual report) to a detailed account of the various modes in which patients were entertained and distracted, including balls, the introduction of pets and visiting children into the wards, and excursions into the city for the theatre and public lectures. All this together, he concluded, amounted to ‘the full development of the system of Non-restraint, Non-seclusion, and exhilarating engagement, in this house …’^80^

## A Microcosm of the Asylum

The *Lancet* editor, Wakley, writing at the end of 1840, reflected back on the debate in his publication’s pages that had brought Hill’s explosive innovation—the abolition of mechanical restraint—to national attention, and thus, unexpectedly, had shone a harsh light on seclusion also. Wakley expressed gratitude to Charlesworth and Hill, while quibbling with their slogan:

The term ‘non-restraint’, we may remark, is not literally correct; for, when the system is most rigidly carried out, the patient is *confined* to the asylum, and in many cases to his room. But this confinement is not felt like fetters; it is less degrading, irritating, and exasperating, than ligatures on the limbs. The restraint is little more severe than the voluntary confinement of servants to the house, or of workmen to their daily task.^81^

The dilemma of seclusion was the dilemma of the asylum in microcosm. The removal of restraints from the limbs of its inmates served to call attention to the forms of restraint represented by the patient’s locked room, and by the enclosing walls of the institution itself. Wakley and other non-restraint supporters were put on the back foot, compelled to offer tortuous justifications. The whole purpose of the asylum—embodied in its very name—was to offer retreat, retirement and removal from the stresses and irritants of life in the world. But that sense of distance and enclosure could easily tip over into a suspicion of imprisonment, invisibility and abuse. At the pivotal moment in asylum history represented by the introduction of non-restraint, the single room behind the locked door—the cell—became a microcosm of this dilemma of symbolism—and also the site of new, very real and highly controversial practices.

## Footnotes

1 Jane Hamlett, with Lesley Hoskins and Rebecca Preston, ‘Introduction’, in *Residential Institutions in Britain, 1725–1970: Inmates and Environments* (London: Pickering & Chatto, 2014), 1–15. Jane Hamlett, *At Home in the Institution: Material Life in Asylums, Lodging Houses and Schools in Victorian and Edwardian England* (Basingstoke: Palgrave Macmillan, 2015).

2 Hamlett, *At Home in the Institution*, 30, 76–78, 80–81, 83. Thomas Crook, ‘Power, Privacy and Pleasure: Liberalism and the Modern Cubicle’, *Cultural Studies*, 2007, 21, 549–69.

3 Michael Donnelly, *Managing the Mind: A Study of Medical Psychology in Early Nineteenth-Century Britain* (London: Tavistock, 1983), 31.

4 Michael Ignatieff, *A Just Measure of Pain: The Penitentiary in the Industrial Revolution, 1750–1850* (Harmondsworth: Penguin, 1989). Ignatieff traces the ideological conflict about the use of solitary confinement back to the John Howard-inspired reform prisons and gaols of the late eighteenth century.

5 Robert Gardiner Hill, *Total Abolition of Personal Restraint in the Treatment of the Insane, a Lect. on the Management of Lunatic Asylums* (London: Simpkin Marshall, 1839). For the role of Charlesworth, Hill and the Lincoln Asylum in the beginnings of the non-restraint movement, see Justin A. Frank Jr., ‘Non-Restraint and Robert Gardiner Hill’, *Bulletin of the History of Medicine*, 1967, 41, 140–60; Leonard D. Smith, ‘The “Great Experiment”: The Place of Lincoln in the History of Psychiatry’, *Lincolnshire History and Archaeology*, 1995, 30, 55–62; Leonard D. Smith, *‘Cure, Comfort and Safe Custody’: Public Lunatic Asylums in Early Nineteenth-Century England* (London: Leicester University Press, 1999), 261–66.

6 John Conolly, ‘Review of Robert Gardiner Hill, Total Abolition’, *British and Foreign Medical Review*, 1840, 9, 153–54. In the period dealt with in this article, Lincoln asylum had around 100 patients; Hanwell had nearly 1,000. Frank, ‘Non-Restraint and Robert Gardiner Hill’, 114; Richard Hunter and Ida Macalpine, ‘Introduction’, in John Conolly, *The Construction and Government of Lunatic Asylums and Hospitals for the Insane*, Psychiatric Monograph Series, 6 (London: Dawsons, 1968), 35. For Conolly and his adoption of non-restraint at Hanwell Asylum, the standard celebratory account is Hunter and Macalpine, ‘Introduction’, Conolly, *Construction and Government*, 21–38. Key revisionist accounts are Andrew T. Scull, *Social Order/Mental Disorder: Anglo-American Psychiatry in Historical Perspective* (London: Routledge, 1989), 187–89 and Akihito Suzuki, ‘The Politics and Ideology of Non-Restraint: The Case of the Hanwell Asylum’, *Medical History*, 1995, 39, 1–17.

7 As is discussed below, Hill included seclusion as a form of restraint, but not as a form that should be abolished. For ‘severity’ see Hill, *Total Abolition*, v.

8 See Smith, *‘Cure, Comfort’*, 248–59. for an account of the prevalence of and implements used in mechanical restraint.

9 Smith, ‘“Great Experiment”’, 58.

10
*The Lancet*, 8 February 1840, 732–33. For Wakley’s role, see Scull, *Social Order*, 190, n. 156.

11 Smith explains the unusual medico-administrative structures at Lincoln: there were three visiting physicians, rather than the usual one, and they served in rotation, a month at a time. In addition, there was a resident ‘house surgeon’ (subordinate to the visiting physicians) and all were overseen by a very active board of governors, places on which could be obtained through donations. Charlesworth, having been physician to the asylum, continued to be highly influential on asylum policy through his role on the board. Hill, after being pushed out as house surgeon, continued to support and advise his successor, and became chair of the board. Hadwen was a former House Surgeon who had subsequently become a Governor. Cookson was a visiting physician. Smith, ‘“Great Experiment”’, 55, 57–9.

12 Kathleen Jones traces the process by which the evidence and recommendations contained in the 1844 report led to national legislation with the Lunatics Act of 1845. Jones, *A History of the Mental Health Services* (London: Routledge & Kegan Paul, 1972), 134–5, 143–9.

13 A Looker On, ‘Letter to the Editor’, *The Lancet*, 24 October 1840, 164–65. See Suzuki, ‘Politics and Ideology’, 10 for the identification of Adams as the anonymous ‘Looker On’.

14 W. D. Cookson, ‘Letter to the Editor’, *The Lancet*, 7 November 1840, 230.

15 Samuel Hadwen, ‘Letter to the Editor’, *The Lancet*, 12 September 1840, 906.

16 Hadwen, 907. Emphasis in the original.

17 Robert Gardiner Hill, ‘Letter to the Editor’, *The Lancet*, 31 October 1840, 198.

18 W. D. Cookson, ‘Letter to the Editor’, *The Lancet*, 28 November 1840, 339; Hadwen, ‘Letter to the Editor’, 906.

19 Cookson, ‘Letter to the Editor’, 7 November 1840, 230.

20 C. C. Corsellis, ‘Letter to the Editor’, *The Lancet*, 21 March 1840, 962. Corsellis’s defensive position should be seen in the context of the legacy of the Wakefield asylum as a model of liberality towards patients, under the previous regime of William Ellis. Corsellis’s time as superintendent rolled back this legacy, reverting to the widespread use of mechanical restraints. John Walton, ‘The Treatment of Pauper Lunatics in Victorian England: The Case of Lancaster Asylum, 1816–1870’, in Andrew Scull, ed., *Madhouses, Mad-Doctors, and Madmen: The Social History of Psychiatry in the Victorian Era* (London: Athlone Press, 1981), 167.

21 Kathleen Jones, *Lunacy, Law, and Conscience, 1744–1845: The Social History of the Care of the Insane*, International Library of Sociology (London: Routledge, 1955), 170–4.

22
*Report of the Metropolitan Commissioners in Lunacy to the Lord Chancellor* (London: Bradbury and Evans, 1844), 137–46. The main author of the report was the prominent reformer Lord Ashley (later the 7th Earl Shaftesbury), but Kathleen Jones argues that he was not responsible for the section on non-restraint, since he had given enthusiastic support to non-restraint and particularly to Conolly, in the past (Jones, *Lunacy, Law*, 181–3), Jones goes on to explain that Thomas Wakley raised the report soon after its publication in the House of Commons, ‘and stated flatly that it was obviously not the work of Ashley’ (p. 183). See also Hunter and Macalpine, ‘Introduction’, in Conolly, *Treatment of the Insane without Mechanical Restraints*, Psychiatric Monographs Series (Folkestone: Dawsons, 1973), xviii, n. 46.

23
*Report of the Metropolitan Commissioners*, 137–8.

24
*Report of the Metropolitan Commissioners*, 141.

25 For a discussion of Conolly’s views on the role of single rooms generally in asylums (including, but not only, as containers for forcible confinement), see Leslie Topp, ‘Isolation, Privacy, Control and Privilege: Psychiatric Architecture and the Single Room’, in Sarah Schrank and Didem Ekici, eds, *Healing Spaces, Modern Architecture and the Body* Ashgate Studies in Architecture (London: Routledge, 2016), 88–92. See also Elaine Showalter, *The Female Malady: Women, Madness, and English Culture, 1830–1980* (London: Virago, 1987), 37.

26 Annual Report of the Physicians to the Visiting Magistrates (Hanwell Asylum), 1840, excerpted in John Conolly, *The Treatment of the Insane without Mechanical Restraints* (London: Smith, Elder, 1856), 207.

27 Conolly, *Treatment of the Insane*, 207–8.

28 Conolly, *Treatment of the Insane*, 208.

29 Conolly, *Treatment of the Insane*, 211.

30 Hill, *Total Abolition*, 49.

31 Appendix C (two letters from William Smith to the Lincoln Asylum Board of Governors), letter dated 25 October 1841 in *State of the Lincoln Lunatic Asylum (Annual Report)*, vol. 18 (Lincoln, 1842), 27.

32
*State of the Lincoln Lunatic Asylum (Annual Report)*, 18:5.

33 Appendix C, letter dated 12 January 1842, in *State of the Lincoln Lunatic Asylum (Annual Report)*, 18:28.

34
*Report of the Metropolitan Commissioners*, 146. See also Smith, *‘Cure, Comfort’*, 273. As Smith has shown, the radical momentum continued at Lincoln, with Charlesworth in 1845–6 questioning, in the name of freedom of movement for patients both inside the asylum building and in the airing grounds, the previously sacrosanct principle of the classification of patients by behaviour. Smith, ‘“Great Experiment”’, 60–1.

35 Christine Stevenson, in her discussion of Robert Hooke’s 1674 building for Bethlem, shows how contemporaries noted its provision of separate cells for each inmate, and suggests that the provision of cells in buildings for the mad went back further, since there is evidence that Bethlem’s first incarnation at Bishopsgate housed its charges in cubicles. Christine Stevenson, *Medicine and Magnificence: British Hospital and Asylum Architecture, 1660–1815* (New Haven, CT: Paul Mellon Centre for Studies in British Art and Yale University Press, 2000), 54. Leonard Smith shows how George Dance’s St Luke’s Hospital in Old Street, London (1787) consolidated ‘the inter-relation of cells and large galleries’ as the standard spatial configuration for asylums. Leonard D. Smith, *Lunatic Hospitals in Georgian England, 1750–1830* (London: Routledge, 2007), 19. The County Asylums Act of 1808, which gave detailed instructions for the construction of county asylums, included the requirement that new asylums contain ‘dry and airy Cells for Lunatics of every Description’, alongside day rooms. Jones, *Lunacy, Law*, 74–6, here 76.

36 Hill, *Total Abolition*, 11.

37 Kathleen Jones, in her discussion of the evolution of lunacy reform from the 1760s onwards, finds multiple references in the primary sources to cells as dark and cramped spaces in which people might languish forgotten for years. For instance, Dr Andrew Halliday reported the discovery in Norfolk and Suffolk of 47 lunatics and 67 idiots confined to ‘damp, dark cells’, previously unacknowledged by the authories in those counties and Godfrey Higgins in his 1814 investigations of conditions in the York Asylum uncovered a series of eight feet square cells ‘in a very horrid and filthy condition’. Jones, *Lunacy, Law*, 73, 87–8 and see also 32, 68, 100–2. As Michael Donnelly reports, visitors were often struck by the physical impact on inmates of such surroundings: ‘In the 1815 Parliamentary investigations one physician described the slow, halting movement toward the light of a barely-sighted, albino-like creature whom he and other asylum inspectors had disturbed in his cell during a visit.’ Donnelly, *Managing the Mind* 33. See also Smith, *Lunatic Hospitals in Georgian Britain*, 172–3.

38 Hill, *Total Abolition*, 69, 79, Appendix B.

39 Hill, *Total Abolition*, 69–70.

40 Hill, *Total Abolition*, 91.

41
*Report of the Metropolitan Commissioners*, 146.

42 Jones, *Lunacy, Law*, 193–4.

43 Conolly, *Treatment of the Insane*, 45–6.

44 Annual Report of the Physicians to the Visiting Magistrates (Hanwell Asylum), 1844, excerpted in Conolly, *Treatment of the Insane*, 253.

45 John Conolly, *The Construction and Government of Lunatic Asylums and Hospitals for the Insane* (London: John Churchill, 1847), 28.

46 Conolly, *Treatment of the Insane*, 26–7.

47 Annual Report of the Physicians to the Visiting Magistrates (Hanwell Asylum), 1841, excerpted in Conolly, *Treatment of the Insane*, 233.

48 Annual Report of the Physicians to the Visiting Magistrtes (Hanwell Asylum), 1841, excerpted in Conolly, *Treatment of the Insane*, 254–55.

49 Showalter, *Female Malady*, 36–37.

50 For the Victorian domestic interior and its associations with the Romantic idyll see Stefan Muthesius, *The Poetic Home: Designing the 19th-Century Domestic Interior*, (London: Thames and Hudson, 2009). The first use of ‘seclusion’ as as noun meaning a condition or state of being secluded and apart from society cited in the OED is from William Cowper’s early Romantic poem *The Task* (1785), iii., 675: ‘Oh blest seclusion from a jarring world’.

51 Hadwen, ‘Letter to the Editor’, 907.

52 William Simpson, ‘Letter to the Editor’, *The Lancet*, 6 June 1840, 370, emphasis in the original. The phrase ‘habits contrary to nature’ refers of course to masturbation. A letter following up on Simpson’s in the next *Lancet* issue was explicit about this, and in fact cited masturbation as the main cause of the mental deterioration prisoners suffered. William Wilson, ‘Letter to the Editor’, *The Lancet*, 13 June 1840, 422. See also Charles Tulk, ‘Letter to the Editor’, *The Lancet*, 21 November 1840, 296. For the 1840s critique of solitary confinement at Pentonville as causing insanity see Robin Evans, *The Fabrication of Virtue: English Prison Architecture, 1750–1840* (Cambridge: Cambridge University Press, 1982), 384–7.

53 Ignatieff, *A Just Measure of Pain*, 102–3, here 102.

54 Ignatieff, *A Just Measure of Pain*, 124–42. A forthcoming article by Catherine Cox and Hilary Marland closely examines the campaign for the establishment of the separate system at Pentonville, the first few years of its operation and the incidence of mental illness among prisoners as a result of long periods of solitary confinement. Cox and Marland, ‘“He must die or go mad in this place”: Prisoners, Insanity and the Pentonville Model Prison Experiment, 1842–1852’, *Bulletin of the History of Medicine*, forthcoming. I am grateful to the authors for sharing their text with me in advance of publication.

55 Annual Report of the Physicians to the Visiting Magistrates (Hanwell Asylum), 1841, excerpted in Conolly, *Treatment of the Insane*, 231.

56
*State of the Lincoln Lunatic Asylum (Annual Report)*, 18:5 and see also 27, 28.

57
*Report of the Metropolitan Commissioners*, 146 and see also 141; Conolly blamed this report for falsely labelling seclusion as solitary confinement. Conolly, *Treatment of the Insane*, 41–2.

58 Hadwen, ‘Letter to the Editor’, 907.

59 Annual Report of the Physicians to the Visiting Magistrates (Hanwell Asylum), 1844, excerpted in Conolly, *Treatment of the Insane*, 255–6.

60 Robert Gardiner Hill, ‘Letter to the Editor’, *The Lancet*, 22 February 1840, 797; see also Conolly, *Treatment of the Insane*, 47.

61 Tulk, ‘Letter to the Editor’, 297. See Suzuki, ‘Politics and Ideology’, 6–11 for Tulk and Adams’ involvement in Hanwell.

62 Tulk, ‘Letter to the Editor’, 297, 296. Ignatieff points to the use of confinement in dark cells as a punishment at Gloucester Prison in the late eighteenth century. Ignatieff, *A Just Measure of Pain*, 103.

63 Smith, *‘Cure, Comfort’*, 272.

64 Charlesworth published his answers to these questions, as they pertained to the Lincoln Asylum in Edward Parker Charlesworth, *Remarks on the Treatment of the Insane, and the Management of Lunatic Asylums, the Substance of a Return from the Lincoln Lunatic Asylum to the Circular of His Majesty’s Secretary of State* (London, 1828). His answer to this question was: ‘Dark solitary rooms have been much used in cases of violent maniacal excitement, and with great advantage’ (p. 17). For the Select Committee report and the link to Charlesworth’s book, see Jones, *Lunacy, Law*, 139–40.

65 Conolly related the use of the darkened room to the exclusion of light (and visitors) from the sick room of a patient with fever: ‘[seclusion] is as much adapted to secure an irritable brain from causes of increased irritability as a quiet chamber and the exclusion of glare, and of many visitors, is adapted to the same state of brain in a fever’. Conolly, *Treatment of the Insane*, 43.

66 Annual Report (Hanwell Asylum), 1844, excerpted in Conolly, *Treatment of the Insane*, 253.

67 Annual Report (Hanwell Asylum), 1840, excerpted in Conolly, *Treatment of the Insane*, 211.

68 Annual Report (Hanwell Asylum), 1841, excerpted in Conolly, *Treatment of the Insane*, 232. Conolly, *Construction and Government*, 28–9.

69 Annual Reports (Hanwell Asylum), 1841, 1844 excerpted in Conolly, *Treatment of the Insane*, 207, 254.

70
*Report of the Metropolitan Commissioners*, 141.

71 Cookson, ‘Letter to the Editor’, 28 November 1840, 339.

72 Hadwen, ‘Letter to the Editor’, 906–7.

73 Corsellis, ‘Letter to the Editor’, 962; see also Arthur Stillwell, ‘Letter to the Editor’, *The Lancet*, 5 December 1840, 371–2.

74 J. C. Bucknill and D. H. Tuke, *A Manual of Psychological Medicine* (London, 1858), 525.

75 For the furnishing of the single bedroom see Conolly, *Construction and Government*, 20–1.

76 Annual Report (Hanwell Asylum), 1844, excerpted in Conolly, *Treatment of the Insane*, 253. See also Conolly, *Construction and Government*, 28, 115. Although Conolly gives the impression in his painstaking description that the room is a recent innovation (possibly his own), devised specifically for use under a non-restraint regime, it does seem that padded cells predated the non-restraint movement. The German psychiatrist Maximilian Jacobi describes a padded or upholstered room in his 1834 guide to the construction of asylums. C. W. M. Jacobi, *Ueber die Anlegung und Einrichtung von Irren-Heilanstalten mit ausführlicher Darstellung der Irren-Heilanstalt zu Siegburg* (Berlin: Reimer, 1834), 80.

77 Conolly, *Treatment of the Insane*, 44–5.

78
*Report of the Metropolitan Commissioners*, 146. Smith records evidence of padded rooms being installed in Hanwell and Gloucester Asylums. Smith, *‘Cure, Comfort’*, 272.

79
*State of the Lincoln Lunatic Asylum (Annual Report)*, 18: 28.

80
*State of the Lincoln Lunatic Asylum (Annual Report)*, 18, 6–10, quotation from p. 10. For the balls, excursions and other social events organised in these years at Lincoln see Frank, ‘Non-Restraint and Robert Gardiner Hill’, 154–5.

81 ‘Editorial’, *The Lancet*, 5 December 1840, 377.

## Notes

Leslie Topp is an architectural historian who has written widely on psychiatric spaces. She co-edited *Madness, Architecture and the Built Environment* (Routledge, 2007) and co-curated the exhibition ‘Madness and Modernity: Mental Illness and the Visual Arts in Vienna 1900’ at the Wellcome Collection in 2009. Her book *Freedom and the Cage: Modern Architecture and Psychiatry in Central Europe, 1890–-1914* was published in 2017 by Penn State University Press.

